# Flexible Bronchoscopy Combined with Rigid Bronchoscopy for Treatment of Scarring in the Bronchus Caused by a Foreign Body

**DOI:** 10.1155/2019/4616298

**Published:** 2019-06-17

**Authors:** Lei Wu, Yuanjian Sheng, Xuefeng Xu, Zhimin Chen, Qiang Wang, Zhifang Wang, Yu Yin

**Affiliations:** ^1^Department of Pulmonology, Children's Hospital, Zhejiang University School of Medicine, Hangzhou, China; ^2^Department of Paediatrics, Shaoxing Keqiao Women & Children's Hospital, Shaoxing, China

## Abstract

Foreign body (FB) aspiration into the tracheobronchial tree is an emergency in the pediatric department, particularly in children aged <3 years. FB granulation tissue is commonly found in children with FB aspiration. However, scarring in the bronchus caused by a FB is rare. We herein report a case involving aspiration of a plastic whistle toy with scarring in the bronchus. The scar tissue was successfully removed by interventional bronchoscopy combined with a flexible electrosurgery probe and carbon dioxide cryotherapy.

## 1. Introduction

Aspiration of a foreign body (FB) into the trachea and bronchus is common among children, especially in infants and young children [1]. Complications including pneumonia, pneumomediastinum, pneumothorax, and pulmonary atelectasis have been reported [2]. If not extracted early, a retained FB can result in an inflammatory response and granulation tissue formation around the object, mucosal erosions, edema, and strictures [3]. In severe cases, surgical intervention is sometimes needed for dislodgement of the FB [2]. However, interventional pulmonology modalities (e.g., bronchoscopy, electrocoagulation, and cryotherapy) may help to reduce the need for surgery [4]. In addition, scarring in the bronchus caused by FB aspiration has rarely been reported [5].

We herein present a rare case of bridging scar tissue formation caused by a FB (a plastic whistle) that resulted in narrowing of the left main bronchus. The FB and scar tissue were removed by interventional pulmonology modalities. The patient's guardians provided written informed consent for the publication of this case report and any accompanying images. Approval for this report was granted by the ethics committee of Children's Hospital, Zhejiang University School of Medicine.

## 2. Case Presentation

**Figure 1:**
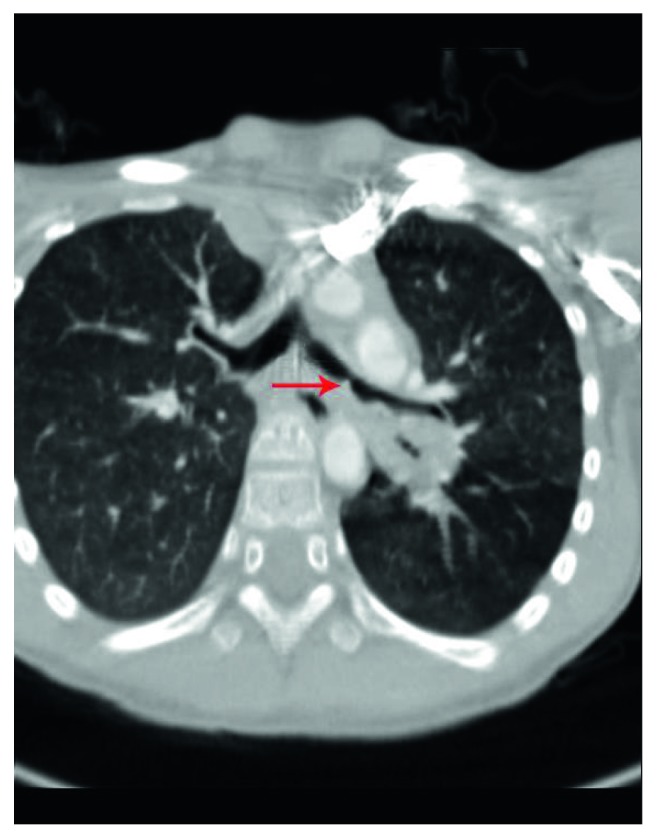
Computed tomography revealed a soft tissue density (red arrow) at the left lower lobe bronchus with narrowing of the left main bronchus and emphysema of the left lower lobe.

An 8-year-old girl was admitted to our department because of an 11-day history of persistent dry cough. Her medical history was not significant. The girl and her parents denied any history of choking or FB aspiration. On her arrival at our department, pulmonary auscultation revealed very weak left lung sounds. Chest computed tomography revealed a soft tissue density at the left lower lobe bronchus (LLLB) with stenosis of the left main bronchus and emphysema of the left lower lobe (Figure 1).

**Figure 2:**
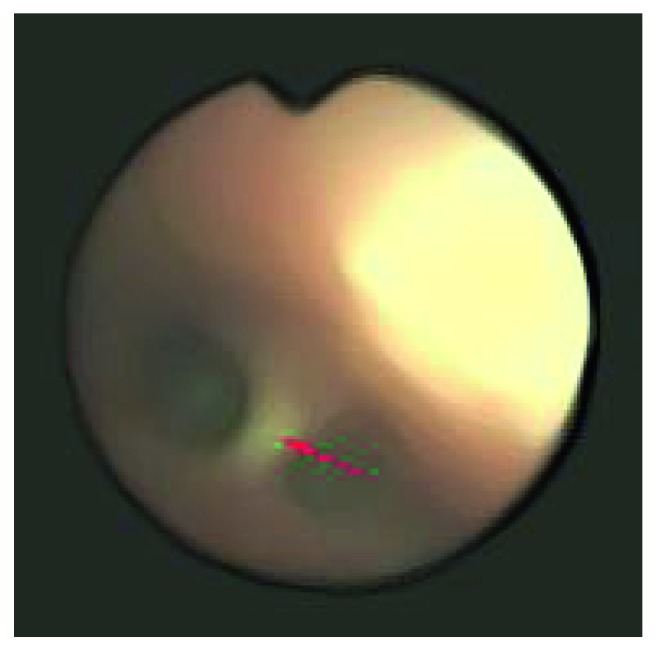
Bronchoscopy revealed stenosis of the left main bronchus caused by bridging scar tissue (red arrow).

Airway exploration by fiberoptic bronchoscopy under sedation and local anesthesia through the nasal route was performed to locate the suspected bronchial lesion. Bronchoscopy revealed that the stenosis of the left main bronchus was caused by a bridging scar tissue (Figure 2). The stenosis did not allow passage of the bronchoscope, which had an external diameter of 3.6 mm. A smaller bronchoscope with an external diameter of 2.8 mm was able to pass through either lumen divided by the bridging scar tissue. Advancement of the bronchoscope revealed that the bridging scar tissue ended 5 mm above the left second carina, and a pink tubular-shaped FB was lodged in the LLLB. However, the nature of the FB in the left main bronchus was unclear. Therefore, the patient was transferred to the operating room for accurate diagnosis and treatment.

**Figure 3:**
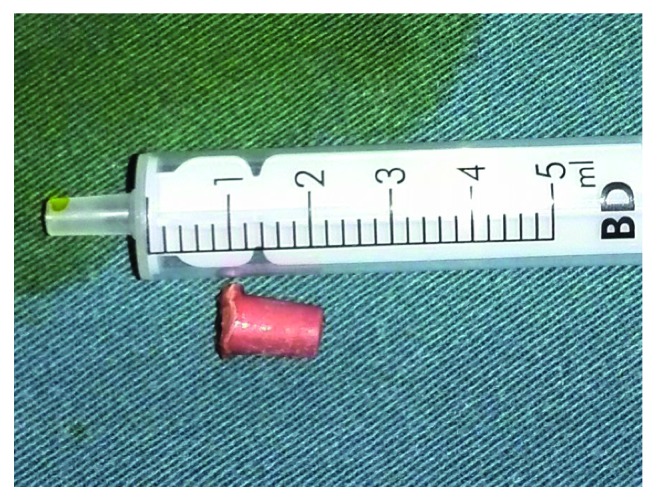
The plastic whistle that had been lodged in the left lower lobe bronchus.

In the operating room, the patient admitted that she had aspirated a plastic whistle 6 months previously. A laryngeal mask was used during general anesthesia, allowing the patient to breathe spontaneously. A swivel adapter was used to connect the proximal end of the laryngeal mask to the T-piece anesthesia system. A flexible fiber bronchoscope (4.9 mm outer diameter) was inserted via the swivel adapter. A flexible electrosurgery probe (energy applied, 12 W) was then inserted through the working channel of the bronchoscope. The target scar was endoscopically visualized and cut by the probe. A grasping forceps was introduced via the suction channel of the bronchoscope to remove the FB. However, lack of sufficient pulling force prevented removal of the FB. A rigid bronchoscope was then introduced, and we successfully removed the plastic whistle from the LLLB using rigid forceps (Figure 3). Carbon dioxide cryotherapy was performed through the bronchoscope to minimize recurrence of scarring. The tip of the cryoprobe was positioned directly on the scar. Freezing was performed for 1 min (at least twice), and the probe was moved until the entire visible lesion had been frozen.

**Figure 4:**
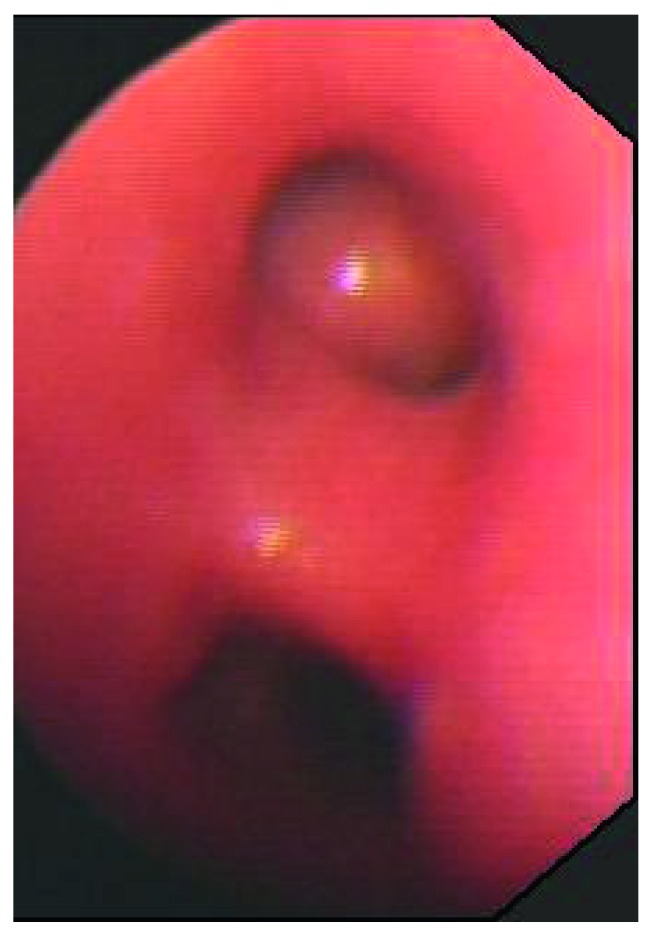
Three months after the surgery, bronchoscopic examination showed good patency of the truncus and the left lower lobe bronchus.

Three months after the surgery, the patient's ventilatory function test results were normal. Bronchoscopic examination showed good patency of the truncus and LLLB (Figure 4).

## 3. Discussion

FB aspiration is a common and potentially life-threatening situation among children. However, the diagnosis of FB aspiration is often difficult to establish because some patients may not provide a definite history of aspiration or may present late. In addition, patients may be misdiagnosed with chronic pneumonia, bronchitis, asthma, or malignancy. Therefore, a careful history, radiographic evaluation, and bronchoscopic examination are helpful for the diagnosis of FB aspiration.

The features of tissue reactions to FBs include granulation tissue formation, endobronchial stenosis, strictures, edema, and airway distortion; in contrast, scarring in the bronchus is rare [5]. The formation of granulation tissue and scarring is highly dependent on the type of FB and the duration of contact with the mucosa. If scars have formed, thoracic surgical interventions are possible. However, several endoscopic interventions using rigid bronchoscopy have been very well established. These include cold knife therapy, balloon dilatation, and intralesional steroid application.

Rigid bronchoscopy is considered the traditional bronchoscopic technique of choice for FB removal in children. It allows excellent control of the airway, provides a large working channel, and permits the use of a wide variety of extraction instruments for removal of FBs and thick mucous plugs. However, a rigid bronchoscope cannot be passed to the distal airways. In addition, visualization of the upper lobe bronchi is difficult. Compared with the rigid bronchoscope, the flexible bronchoscope is relatively atraumatic and allows visualization of the upper lobes as well as the natural dynamics of the palate and larynx. The laryngeal mask airway is a safe and effective adjunct to fiberoptic bronchoscopy under general anesthesia in children. Its larger internal diameter than that of a tracheal tube permits the use of a relatively large fiberoptic bronchoscope without a significant increase in airway resistance [6].

Accumulated data have shown the promising value of flexible bronchoscopy in the extraction of airway FBs. In our department, FBs were removed by flexible bronchoscopy with a disposable grasping forceps or biopsy forceps with a high success rate of 90% [7]. The advantages of initial flexible bronchoscopy include cost-effectiveness, usefulness, and safety [7]. Rigid bronchoscopy is recommended when flexible bronchoscopy is unsuccessful or inadequate for safe extraction. Moreover, if the FB is wrapped by granulation or scar tissue or is difficult to grasp with flexible forceps because of its size or shape, rigid bronchoscopy should be used for extraction. In the present study, the diameter of the whistle was larger than the narrowed left main bronchus, and the whistle could not be removed under direct visualization by an optical grasper with a telescope via a flexible bronchoscope. When the flexible bronchoscope failed to pull the FB out, an electrotome was used to eliminate the scar tissue before FB removal, and a rigid bronchoscope was then used to successfully remove the FB.

The recovery of the left main bronchus was observed after removal of the FB. Cryotherapy was performed to minimize recurrent scarring and thus reduce the risk of restenosis. Cryotherapy involves insertion of a cryoprobe through the working channel of the bronchoscope to create a freeze-thaw effect on the target tissue. This process induces coagulation necrosis of the selected tissue and destruction of the lesion [8]. Cryotherapy has been used to treat malignant and benign airway tumors and remove granulation tissue, FBs, and blood clots. Because of its selective tissue destruction, cryotherapy is less likely to affect the cartilage, collagen, or fat tissues in the airway; thus, the risk of perforation is low [9]. Compared with laser therapy, which is commonly used to treat tracheal stenosis through a rigid bronchoscope, cryotherapy is less likely to cause hemorrhage or perforation, is less expensive, and is relatively simple to perform [8].

Our case demonstrates that bronchoscopy with multiple modalities (e.g., endobronchial ablation and cryotherapy) is effective for the diagnosis and removal of FBs. Similarly, Fang et al. reported that flexible bronchoscopy with different modalities (including a forceps, loop, basket, knife, electromagnet, and cryotherapy) showed a high success rate (90%) in removing FBs [10]. Collectively, these data indicate that endobronchial ablation and cryotherapy techniques may be necessary when FB retention has caused significant scars.

## 4. Conclusion

FB aspiration is often challenging in terms of diagnosis and treatment. Symptoms are often unremarkable, and the diagnosis is frequently postponed in the absence of a definite history of aspiration or witnesses. Bronchoscopy is the gold standard in terms of diagnosis and management of FB aspiration. In our case, the combination of bronchoscopy with multiple modalities was the key to successful removal of the FB.
